# Effect of Spatial Inhomogeneity on Quantum Trapping

**DOI:** 10.1021/acs.jpclett.2c00807

**Published:** 2022-05-16

**Authors:** Victoria Esteso, Sol Carretero-Palacios, Hernán Míguez

**Affiliations:** † Institute of Materials Science of Seville, Consejo Superior de Investigaciones Científicas (CSIC), Universidad de Sevilla (US), Américo Vespucio 49, 41092 Seville, Spain; ‡ Departamento de Física de Materiales, Instituto de Materiales Nicolás Cabrera, Universidad Autónoma de Madrid, 28049 Madrid, Spain

## Abstract

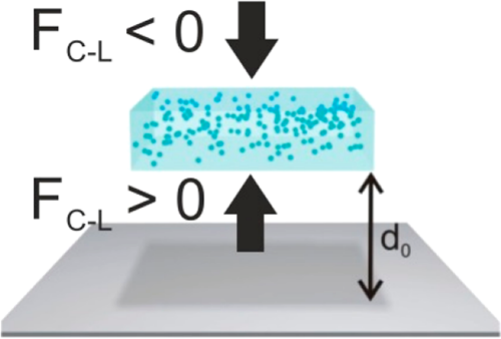

An object that is immersed in a fluid and approaching a substrate may find a potential energy minimum at a certain distance due to the balance between attractive and repulsive Casimir–Lifshitz forces, a phenomenon referred to as quantum trapping. This equilibrium depends on the relative values of the dielectric functions of the materials involved. Herein, we study quantum trapping effects in planar nanocomposite materials and demonstrate that they are strongly dependent on the characteristics of the spatial inhomogeneity. As a model case, we consider spherical particles embedded in an otherwise homogeneous material. We propose an effective medium approximation that accounts for the effect of inclusions and find that an unprecedented and counterintuitive intense repulsive Casimir–Lifshitz force arises as a result of the strong optical scattering and absorption size-dependent resonances caused by their presence. Our results imply that the proper analysis of quantum trapping effects requires comprehensive knowledge and a detailed description of the potential inhomogeneity (caused by imperfections, pores, inclusions, and density variations) present in the materials involved.

Under certain conditions, a moving object immersed in a fluid and approaching a flat substrate may find a potential energy minimum at a distance determined by the balance between the long-range attractive and short-range repulsive Casimir–Lifshitz forces, *F*_C-L_, a phenomenon that is referred to as quantum trapping. If this interaction occurs in the presence of a gravitational field, it gives rise to quantum levitation. These phenomena have been extensively investigated both theoretically^1−10^ and experimentally.^11,12^ The Casimir–Lifshitz interaction, and thus the corresponding trapping equilibrium distance (*d*_eq_), strongly depends on the relative values of the imaginary part of the dielectric functions of the materials composing the system.

In the case of metals, graphene, or complex geometries, including gratings and corrugated surfaces, tackling the Casimir–Lifshitz interaction analytically becomes a complex task due to the spatial dependence of the corresponding dielectric functions.^13−18^ In composites that combine dielectrics and metals, the dielectric function has typically been described using average effective medium models,^19−22^ such as Maxwell–Garnett, Bruggeman, Cuming, and other approximations, which, in most cases, neglect the size of the inhomogeneity and assume it to be arbitrarily small. In doing so, scattering processes that occur inside the material due to porosity, roughness, or the presence of impurities or inclusions, among others, are ignored and their effects on the resulting *F*_C-L_ are overlooked.

In this work, we study the influence, on both *F*_C-L_ and the quantum trapping distance, of single and multiple scattering effects that result from the presence of spatial inhomogeneity inside composite materials. Our model describes the optical behavior of a planar dielectric thin film that contains nanospherical inclusions and is immersed in a fluid by means of a Monte Carlo approach, which integrates Fresnel coefficients, and scattering Mie theory. The complex effective dielectric permittivity () of the inhomogeneous material is then extracted in a reverse process using an oscillatory model that fits the optical characteristics of the composite material. The resulting value of *ε*_eff_(ω) is employed to calculate *F*_C-L_ when the object approaches a substrate. Results demonstrate that the same amount of material distributed in different ways gives rise to different Casimir–Lifshitz interactions, with a subsequent effect on the trapping distance. Our work reveals the need for a proper description of Casimir–Lifshitz interactions between composite materials that accounts for the specific photon resonances their components hold, hence questioning the implementation of standard average medium approximations.

Let us consider a plane-parallel system consisting of an inhomogeneous thin film immersed in glycerol near a silicon (Si) substrate (see the schematic in Figure 1a). The Casimir–Lifshitz interaction between the thin film and the substrate is given by the following expression:^23−25^
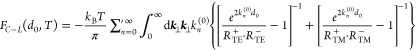
1where *T* is the temperature of the system at thermal equilibrium and *k*_*B*_ is the Boltzmann constant. In addition, ***k***_⊥_ = (*k*_*x*_, *k*_*y*_) and *k*_*n*_^(0)^ account for the components of the wavenumber inside the liquid medium, the subscript *n* = 0, 1, 2, ... describes the discrete and infinite Matsubara frequencies *ξ*_*n*_ = (2*πk*_*B*_*T*)*n*/*ℏ*, and *R*_TE_^±^*R*_TM_^±^ are the multiple Fresnel coefficients for the TE and TM polarizations, respectively, which depend on the values of the dielectric functions of the materials evaluated at *ξ*_*n*_ (further details on this expression are provided in the Supporting Information). In the summation, the “prime” indicates that the *n* = 0 term must be multiplied by a factor 1/2.

**Figure 1:**
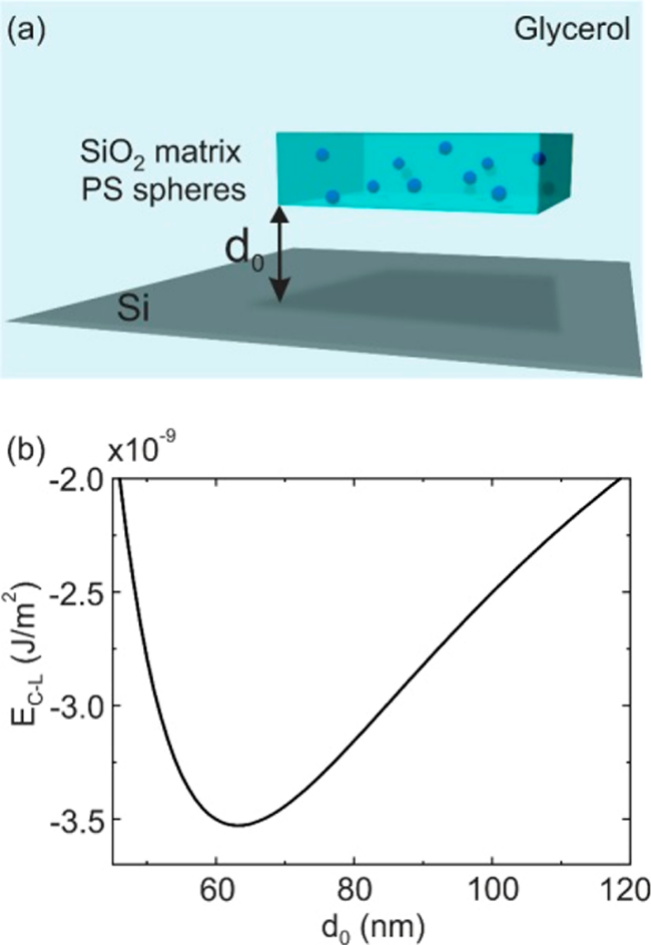
(a) Scheme of two plane-parallel bodies interacting through a fluid. The separation distance between the infinite surfaces is denoted by *d*_0_. One of the bodies is a composite in which spherical PS particles, of radius *r* and volume concentration *ff*, are embedded in an otherwise homogeneous SiO_2_ material. The other interacting material is a Si wafer. The whole system is immersed in glycerol. (b) Exemplary results of the Casimir–Lifshitz potential energy per unit area obtained using the Maxwell–Garnett effective medium model to describe the optical characteristics of a 1000 nm thick SiO_2_ film with *ff* = 10% PS inclusions immersed in glycerol over a Si substrate.

In the particular case studied here, the composite thin film is comprised of two materials that, under identical conditions, display *F*_C-L_ values of similar intensity and opposite sign^7^ when interacting through the glycerol with the Si substrate. Specifically, a silicon dioxide (SiO_2_) matrix with nanospherical polystyrene (PS) inclusions of radius *r* and a concentration expressed through the volume filling fraction (*ff*) is considered. The corresponding complex dielectric permittivities of all materials can be obtained in refs (26−31). To calculate the value of *ε*_eff_(ω) for our inhomogeneous material, first we employ a Monte Carlo approach that combines Fresnel coefficients at the interface between two adjacent layers and scattering Mie theory^32^ to evaluate the scattering (*σ*_S_) and absorption (*σ*_A_) cross sections in an external absorbing medium.^33,34^ This approach, which was described in detail in ref (35) and was used to characterize the optical properties of disordered materials without employing any mixing formula,^36−39^ provides the reflectance (*R*), the absorptance (*A*), and the transmittance (*T*) of the system by tracking the trajectory of a large number of photons impinging on the system. To ensure that there is no correlation between consecutive scattering events, the particle concentration is limited to *ff* < 20% in our model. Next, in a separate procedure, we calculate *R*, *A*, and *T* using the transfer matrix method (TMM)^40^ by assuming a homogeneous thin film whose *ε*_eff_(ω), described by a fitted Drude–Lorentz oscillator model, minimizes the difference between the optical properties attained with the two approaches. Equation 2 displays the general expression of the Drude–Lorentz model applied to adjust the optical characteristics of the composite material, which has been widely used in the calculation of *F*_C-L_ with homogeneous media.^25,31,41−44^
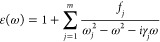
2

In the above expression, *m* is the number of oscillators, *j* is the index of each oscillator, *f*_*j*_ is the oscillator strength, *ω*_*j*_ is the oscillator frequency, and *γ*_*j*_ is the damping parameter. To simplify the fitting process over a wide spectral range (up to several micrometers), the number of oscillators and their spectral positions are fixed to those that fit ε(ω) of the bulk SiO_2_ and PS materials^26−29^ (further details are provided in the Supporting Information). Therefore, to find the best fit of the inhomogeneous material to the optical response, only *f*_*j*_ and *γ*_*j*_ are varied. Finally, to calculate the Casimir–Lifshitz interaction (i.e., the potential energy, *E*_C-L_, or the force, *F*_C-L_) at thermal equilibrium at *T* = 298 K, the expression developed by Lifshitz et al.^23,24^ for two arbitrary planar bodies interacting through a third material was employed. To do so,  attained from eq 2 is transformed to Matsubara frequencies after a Wick rotation:
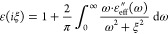
3

Please note that, in order to use Lifshitz equation, our composite film was treated as a homogeneous slab with a fitted effective dielectric constant that accounted for the effect of inclusions.

For illustrative purposes, results obtained using the Maxwell–Garnett effective medium approximation^46^ for a composite dielectric material are shown in Figure 1b. Specifically, Figure 1b shows *E*_C-L_ as a function of the separation distance (*d*_0_) between a composite thin film (a 1000 nm thick SiO_2_ film containing *ff* = 10% PS spherical inclusions) and a substrate, all of which are immersed in glycerol. As it has been shown before, the description of the optical properties of the inhomogeneous material based on the Maxwell–Garnett effective medium approximation predicts the quantum trapping of the system with a well-defined equilibrium distance.^7^

Computing the optical characteristics of the composite material while considering scattering effects requires, as first step, the evaluation of the absorption and scattering cross-section of single dielectric inclusions. To do so, we employed a Mie formalism,^32^ which was modified to account for the presence of losses in the embedding medium.^33,34^ Panels a and b in Figure 2 depict *σ*_A_ and *σ*_S_, respectively, as a function of the incident light wavelength (λ) for different PS nanosphere sizes (from *r* = 10 to 100 nm) embedded into an absorbing SiO_2_ matrix. The curves for *r* = 20, 50, and 100 nm are highlighted, as they will be taken as representative examples of small, medium, and large particle sizes, respectively, in the following analysis. As expected, PS inclusions absorb light very efficiently in the λ < 250 nm wavelength range, the region in which bulk PS strongly absorbs.^28,29,47^ The intensities of those peaks increase with the particle size, and the spectral shape is maintained. Conversely, *σ*_S_ in panel (b) shows that the scattering cross-section becomes larger as the inclusions become larger, but a red-shift of the maximum scattering peak for the largest nanospheres occurs. Please note that for the smallest particles considered *σ*_A_ ≫ *σ*_S_, while for the larger ones *σ*_*A*_ ≈ *σ*_*S*_. This difference has relevant repercussions when the absorption properties of the different composites considered are compared, as will be shown next.

**Figure 2:**
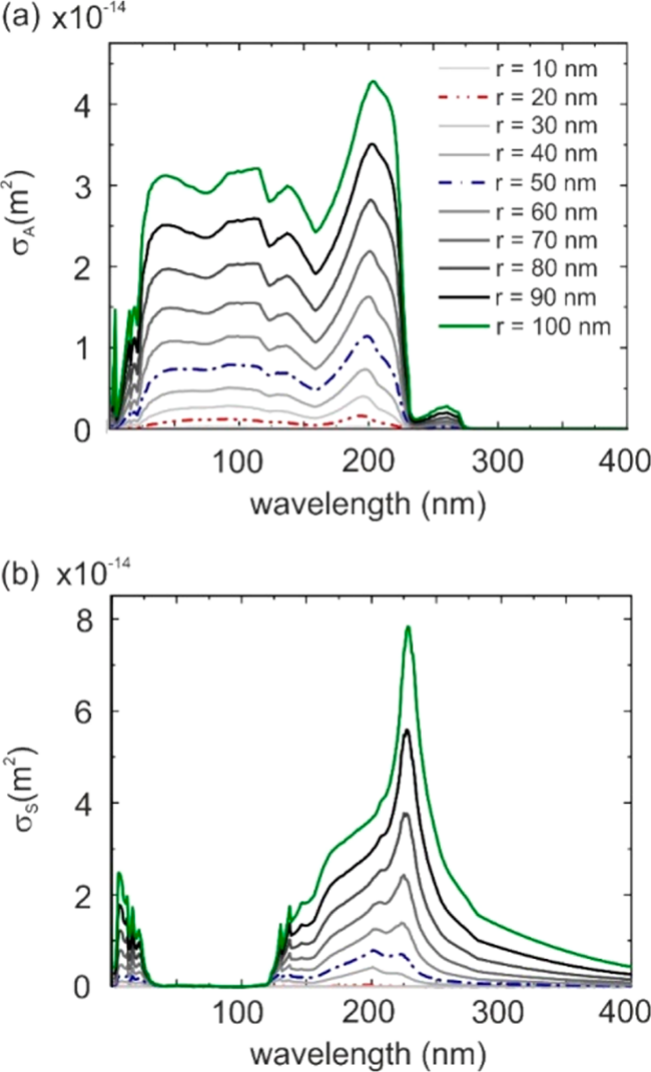
(a) Absorption cross-section, σ_A_, and (b) scattering cross-section, σ_S_, as a function of the incident wavelength of PS inclusions of radius *r* = 10–100 nm (dark increase gray scale lines) embedded in an absorbing SiO_2_ matrix. The curves for *r* = 20, 50, and 100 nm are highlighted in red, blue, and green, respectively, as they will serve as exemplary cases in the analysis in following figures.

The absorption properties of exemplary composite materials and the corresponding effective dielectric permittivities in ω and Matsubara frequencies,  and *ε*_*eff*_(*iξ*), respectively, are shown in Figure 3. Note that, despite the fact that the largest variations in the optical absorption of the composite materials are attained within the spectral range λ ∈ [0, 400] nm, the optical characteristics for wavelengths up to several microns were computed, as this was required to evaluate the Casimir–Lifshitz force. Figure 3a and b displays total absorptance spectra of a 1000 nm thick composite film for various *r* and *ff* values; dilute systems (*ff* < 20%) were assumed to discard possible correlation effects. In both panels, the colored lines correspond to calculations obtained using a Monte Carlo approach,^35−39^ whereas the dashed gray lines refer to absorptance spectra fitted through the Drude–Lorentz model in which the  values of an equivalent homogeneous slab were considered. The excellent match of these curves shows that the absorption properties of an optically disordered thin film can be accounted for by an effective imaginary part of the dielectric function, a necessary condition to apply this approach to the calculation of Casimir–Lifshitz forces. Figure 3a shows results for *r* = 20, 50, and 100 nm PS nanospheres at a fixed concentration in the SiO_2_ matrix. Indistinguishable high absorption bands are attained at shorter wavelengths, λ < 150 nm, at which both SiO_2_ and PS absorb strongly. At λ ≈ 200 nm, bulk SiO_2_ does not absorb and hence all absorption in the composite must be solely attributed to PS inclusions. It can be seen that, for a fixed volume-filling fraction, the inclusion of larger particles gives rise to a smaller absorptance values in spite of their larger absorption cross sections, as shown in Figure 2. This is the result of two effects. First, a much lower particle number density is attained for larger particles, which results in a smaller probability of a photon-inclusion encounter event occuring. Second, the fact that *σ*_*A*_ ≈ *σ*_*S*_ for large particles, which implies that scattering is as likely to occur as absorption when one of these encounters take place. However, in the case of small particles *σ*_*A*_ ≫ *σ*_*S*_, and therefore absorption is favored over scattering. A detailed analysis of the scattering and absorption events that occur at λ = 200 nm for different particle sizes can be found in the Supporting Information. Interestingly, for all particle sizes considered, the fraction of diffusively transmitted or reflected light is always lower than 12% and almost zero for *r* ≤ 40 nm. This result further supports the validity of our approximation based on the description of the inhomogeneous film as an effective homogeneous slab, where the absorption properties (i.e., ) are those mainly modified by the presence of inclusion size-dependent resonances.

**Figure 3:**
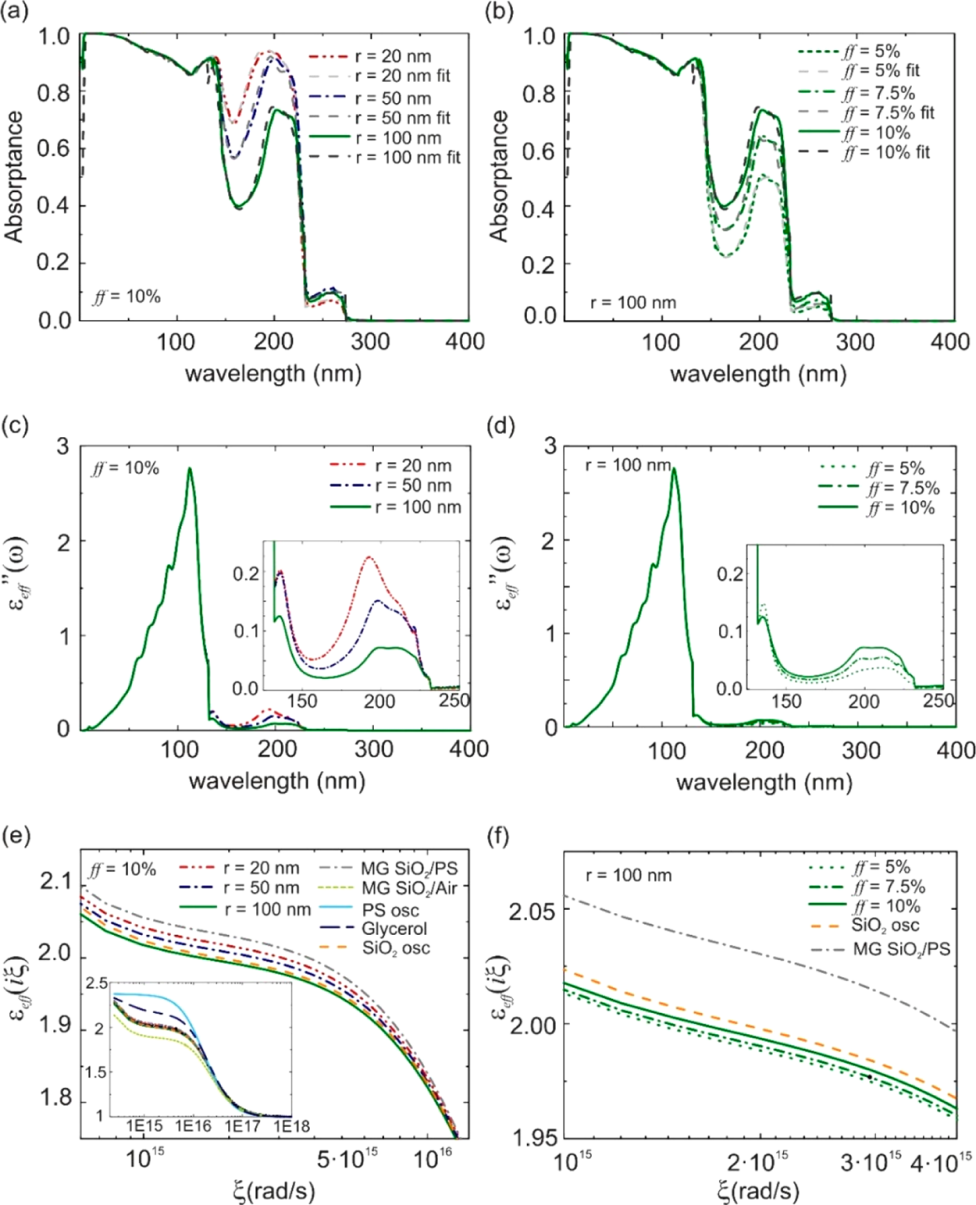
(a and b) Absorptance of a 1000 nm SiO_2_ film with PS inclusions immersed in glycerol. In panel a, *ff* = 10% and the PS radii take the values *r* = 20, 50, and 100 nm, (red, blue, and green lines, respectively). Black, dark gray, and light gray dashed lines account for the corresponding absorptance obtained from the fitting parameters in eq 2. In panel b, *r* = 100 nm and the concentration of PS scattering is *ff* = 5%, 7.5%, and 10% (dotted, dashed dottted, and solid green lines, respectively). Black, dark gray, and light gray dotted lines represent the corresponding absorptance obtained from the fitting parameters in eq 2. (c and d) Imaginary part of the fitted dielectric function evaluated in ω frequencies that reproduce corresponding optical characteristics of the systems in panels a and b. (e and f) Dielectric function evaluated at Matsubara frequencies using the complex part of the fitted effective dielectric functions in panels c and d, respectively. Dashed orange and blue solid lines stand for bulk SiO_2_ and PS materials, respectively. In addition, dashed dotted gray and short dashed green lines depict the dielectric function estimated with the Maxwell–Garnett effective medium model for a SiO_2_ matrix with a 10% PS and void inclusions, respectively. The inset in panel e shows a zoomed-out view of the curves.

The effect of varying the concentration of particles for a fixed particles size was also considered. In Figure 3b, *r* is fixed to 100 nm and *ff* takes values of 5%, 7.5%, and 10%. In this case, as the particle number density of the absorbing PS material increases, an absorption enhancement at λ ≈ 200 nm occurs for larger particle concentrations. The curves of  attained after fitting the corresponding absorptance spectra are plotted in Figure 3c and d. The insets show a zoomed-in view at λ ≈ 200 nm, where the main differences between the curves can be found. Clearly, composite materials containing either larger inclusions at a fixed concentration or fewer nanospheres at a constant particle size results in smaller  values and hence lower absorption. Panels e and f in Figures 3 present the corresponding *ε*_eff_(*iξ*), i.e., the imaginary part of the dielectric constant expressed in Matsubara frequencies after the Wick rotation is employed. For comparison, results of calculations that applied the Maxwell–Garnett effective medium model to describe the optical properties of a thin SiO_2_ film with *ff* = 10% for either PS or void inclusions are also plotted (dashed dot gray and greenish lines, for PS and void inclusions, respectively). Additionally, ε(*iξ*) curves of the bulk SiO_2_ and PS materials are also shown as references. The inset in Figure 3e shows a zoomed-out view of the figure. It can be readily seen that once the effect of the size-dependent resonances of the inclusions is accounted for, the attained *ε*_*eff*_(*iξ*) curves present substantial differences compared to those estimated with the Maxwell–Garnett effective medium model. The effect on *ε*_eff_(*iξ*) of increasing the particle concentration (which would give rise to multiple scattering events) is less relevant than that of enlarging the particle size, which highlights the importance of single scattering events in diluted systems for tuning the optical properties of composite materials. The corresponding effect on *F*_C-L_ and *d*_eq_ is shown in the following section.

Finally, Figure 4 presents the absolute value (on a logarithmic scale) of *F*_C-L_ between a Si substrate and a 1000 nm thick in homogeneous film, with various sets of *r* and *ff* values, in glycerol as a function of the separation distance *d*_0_. For comparison, *F*_C-L_ calculated assuming the effective dielectric function estimated with the Maxwell–Garnet effective medium approximation for either PS inclusions or void space at a concentration *ff* = 10% is also considered. In addition, *F*_C-L_ for a homogeneous SiO_2_ film is also shown. In all cases, the interaction between the immersed film and the substrate is repulsive at short separation distances and attractive at large *d*_0_. In Figure 4a, *ff* is fixed to 10%, and the effect of the particle size is analyzed (*r* = 20, 50, and 100 nm). In Figure 4b, the effect of gravity and hence buoyancy is also included. In Figure 4c, the response of the system to different particle concentrations is studied for PS inclusions of *r* = 100 nm. Interestingly, *F*_C-L_ and, consequently, *d*_eq_ strongly depend on the radii of the inclusions considered. Homogeneous thin films made of PS present solely attractive forces. Therefore, it could be expected that adding PS nanospheres to a homogeneous SiO_2_ matrix would introduce an attractive contribution to *F*_C-L_, hence bringing the trapping object to shorter separation distances as predicted by the Maxwell–Garnett effective medium approach for a SiO_2_ matrix containing PS inclusions. However, although this behavior still holds for small inclusions within our model, we observe that the trapping distance (which is invariably larger than that estimated by the Maxwell–Garnett effective medium model) increases with the size of the inclusions, even exceeding that predicted for a homogeneous SiO_2_ film (d_eq_ = 80 nm) in the case of very large radii (*r* = 100 nm). This counterintuitive result can be ascribed to the outcome of absorption modifications due to single and multiple scattering effects taking place inside the composite material, which adjust *ε*_eff_(*iξ*) and *F*_C-L_ acting on the system^47^ and therefore the quantum trapping distance. In contrast, according to results calculated using the Maxwell–Garnett effective medium approximation, porous SiO_2_ matrixes containing void pores find the equilibrium position at much larger distances. Results for a homogeneous SiO_2_ film further emphasize the unusual nature of the amplification of the Casimir–Lifshitz repulsion due to the correct consideration of the inhomogeneity. In all cases, when gravity is taken into account (Figure 4b), the equilibrium distance is further reduced (as there is an additional attractive component), although this effect is minor. Finally, our model predicts that, for the particle number density range under study, *F*_C-L_ is much less sensitive to changes in concentration for a fixed inclusion size, becoming only slightly more attractive as the concentration increases, as shown in Figure 4c. Our results clearly indicate that size-dependent scattering and absorption events that occur inside composite materials due to the presence of spatial inhomogeneity determine the optical response of the material and have a strong effect on both the Casimir–Lifshitz interaction and quantum trapping distances.

**Figure 4:**
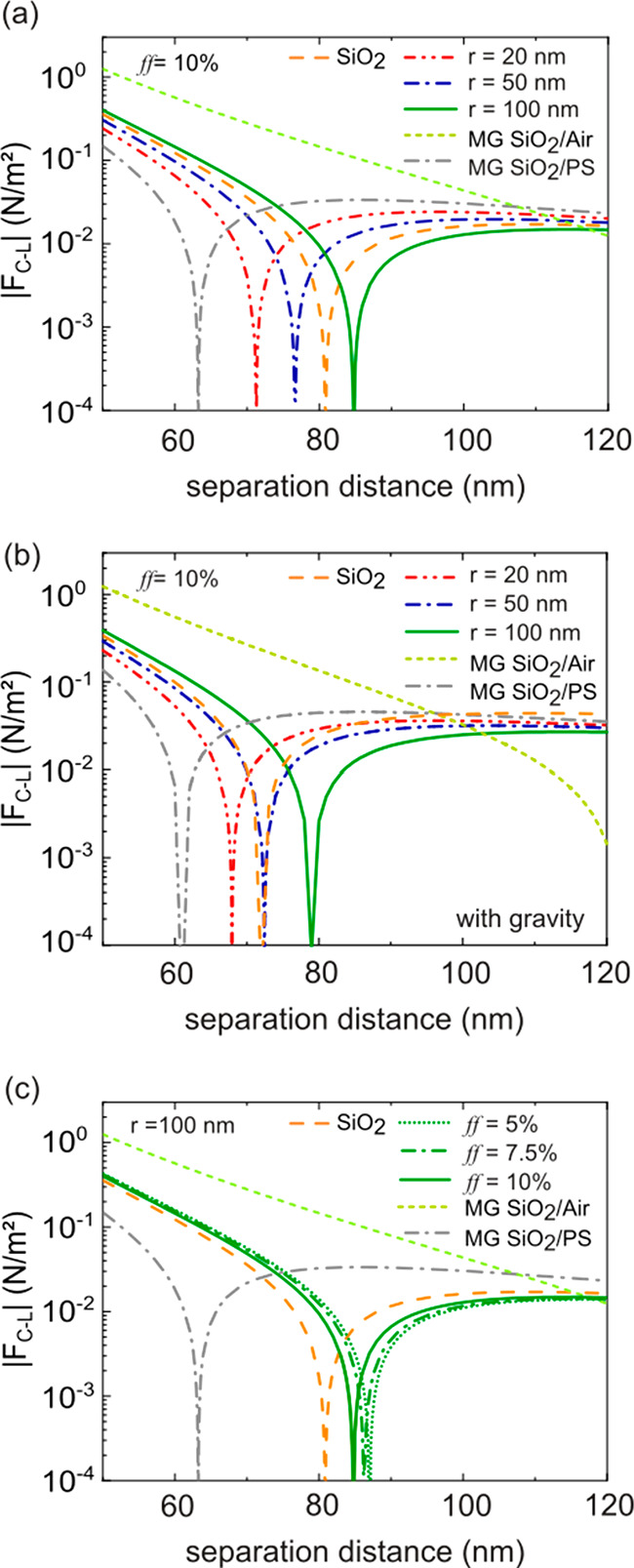
(a) The Casimir–Lifshitz force absolute value between a silicon substrate and a SiO_2_ matrix with PS inclusions, where *r* = 20, 50, and 100 nm (red, blue, and green lines, respectively) and *ff* = 10%, immersed in glycerol as a function of *d*_0_. (b) The same results as those in panel a with the contribution of the gravity force (including buoyancy). (c) The Casimir–Lifshitz force absolute value between a silicon substrate and a SiO_2_ matrix with PS inclusions, where *r* = 100 nm and *ff* = 5%, 7.5%, and 10%, (dotted, dashed dotted, and solid green lines, respectively) as a function of *d*_0_. For comparison, *F*_*C*–*L*_ for a 1000 nm thick composite material is shown in all panels. *F*_*C*–*L*_ was calculated using the Maxwell–Garnett effective medium model by considering both a SiO_2_ matrix with a 10% either PS (dashed dotted gray lines) or void (short dashed greenish line) inclusions and a bulk SiO_2_ film (dashed orange lines).

We have shown that quantum trapping is strongly dependent on the characteristics of the potential spatial inhomogeneity present in the system (porosity, impurities, nanoinclusions, etc.). Our model proposes an effective medium approximation generated from the optical properties of the composite material that accounts for the specific photon resonances its components hold. By considering an experimentally doable model case based on a SiO_2_ matrix embedded with PS nanospheres of different sizes and concentrations (materials that in bulk homogeneous films display forces of opposite sign when approaching the substrate of choice), we find that dominant single-scattering effects yield counterintuitive intense repulsive Casimir–Lifshitz forces that are strongly inclusion size-dependent, calling into question average effective medium models that ignore particle size effects. These results provide a more accurate description of quantum trapping effects in real materials in which inhomogeneity is almost unavoidable and also offer novel alternative means of controlling the separation distances between micro- and nanometer-scale components in devices by means of the rational design of such inhomogeneity. Furthermore, our results are not only important in the framework of Casimir–Lifshitz force but also are of relevance in the fields of photonics and materials science, as the tool we have developed allows estimating the optical properties of all kinds of composites considering the presence of inhomogeneities.

## Supporting Information Available

Details on the computational methods, analysis of the dielectric functions of bulk SiO_2_ and PS, the Maxwell–Garnet effective medium model approach, and scattering and absorption events occurring at λ = 200 nm for different particle sizes (PDF)
