# Supplementary material for: Effect of Spatial Inhomogeneity on Quantum Trapping
Source: J Phys Chem Lett. 2022 May 16;13(20):4513–9. doi: 10.1021/acs.jpclett.2c00807 (PMC9150094; doi:10.1021/acs.jpclett.2c00807)
Supplement: Supplementary file 1 — jz2c00807_si_001.pdf [file jz2c00807_si_001.pdf]

Supporting Information:

Effect of Spatial Inhomogeneity on Quantum Trapping

*Victoria Esteso[1], Sol Carretero-Palacios[2], Hernán Míguez[1,]**

1. Institute of Materials Science of Seville, Consejo Superior de Investigaciones Científicas (CSIC)- Univesidad de Sevilla (US). Américo Vespucio 49, 41092, Seville, Spain

2. Departamento de Física de Materiales, Instituto de Materiales Nicolás Cabrera, Universidad Autónoma de Madrid, 28049-Madrid, Spain

Corresponding Author

*h.miguez@csic.es

1. Formalism for the Casimir-Lifshitz force calculations

The system schematized in Fig. 1(a) is devised as a multilayer structure in which the glycerol medium mediates the interaction between the silicon substrate and the levitating composite. The former consists of an inhomogeneous SiO_2 thin film embedding PS nanoinclusions. In our formalism, each layer in the multilayer structure is indexed by m = -1, 0, 1, and 2, which correspond to silicon, glycerol, composite and glycerol materials, accordingly. The thickness of each layer is denoted by d_m, and the corresponding permittivity evaluated at Matsubara frequencies is given by $\varepsilon^{(m)}(i\xi_n)$. The F_{C-L} is calculated employing the Lifshitz's theoretical formalism expressed for an arbitrary layered system in which the electromagnetic field and the material bodies are treated macroscopically [23-25]:

$$F_{C-L}(d_0,T) = -\frac{k_BT}{\pi}\sum_{n=0}^{\prime\,\infty}\int_0^\infty d\boldsymbol{k}_\perp\ \boldsymbol{k}_\perp\ k_n^{(0)}\left\{\left[\frac{e^{2k_n^{(0)}d_0}}{R_{TE}^{+}\cdot R_{TE}^{-}}-1\right]^{-1}+\left[\frac{e^{2k_n^{(0)}d_0}}{R_{TM}^{+}\cdot R_{TM}^{-}}-1\right]^{-1}\right\} \quad (1)$$

In the above expression, T is the temperature of the system at thermal equilibrium, and k_B the Boltzman constant. In addition, $\boldsymbol{k}_\perp = (k_x, k_y)$ and $k_n^{(0)}$ account for the components of the wavenumber inside the liquid medium, with the subscript $n = 0, 1,2, \ldots$ describing the discrete and infinite Matsubara frequencies $\xi_n = (2\pi k_B T)n/\hbar$. The "prime" in the summation indicates that the term $n = 0$ must be multiplied by a factor 1/2. Also, the multiple Fresnel coefficients for transverse magnetic and transverse electric polarizations, $j = TE, TM$, take the form:

$$R_j^{(\pm)}(n,\ \boldsymbol{k}_\perp) = \frac{r_j^{(0,\pm1)} + r_j^{(\pm1,\pm2)}e^{-2k_n^{(\pm1)}d_{\pm1}}}{1 + r_j^{(0,\pm1)}\cdot r_j^{(\pm1,\pm2)}e^{-2k_n^{(\pm1)}d_{\pm1}}} \quad (2)$$

With

$$r_{TM}^{(m,m')}(n,\ \boldsymbol{k}_\perp) = \frac{\varepsilon_0^{(m')}k_0^{(m)} - \varepsilon_0^{(m)}k_0^{(m')}}{\varepsilon_0^{(m')}k_0^{(m)} + \varepsilon_0^{(m)}k_0^{(m')}} \quad (3)$$

and

$$r_{TE}^{(m,m')}(n,\ \boldsymbol{k}_\perp) = \frac{k_0^{(m)} - k_0^{(m')}}{k_0^{(m)} + k_0^{(m')}} \quad (4)$$

the simple Fresnel coefficients evaluated at Matsubara frequencies, and $k_n^{(m)}$ taking the form:

$$k_n^{(m)} = \left[\boldsymbol{k}_\perp^2 + \varepsilon_n^{(m)}\frac{\xi_n^2}{c^2}\right]^{\frac{1}{2}} \quad (5)$$

Finally, the permittivity evaluated at Matsubara frequencies is given by

$$\varepsilon_n \equiv \varepsilon(i\xi_n) = 1 + \frac{2}{\pi}\int_0^\infty \frac{\omega\varepsilon''(\omega)}{\omega^2 + \xi_n^2}d\omega \qquad (6)$$

with $\varepsilon''(\omega)$ the imaginary part of the complex permittivity at ω frequencies, $\varepsilon(\omega) = \varepsilon'(\omega) + i\varepsilon''(\omega)$.

The total force per unit area acting on the levitating film is given by:

$$F = F_{C-L} + F_g + F_B \quad (10)$$

With F_g the gravity force ($F_g = -gD_1d_1 = -gD_{film}d_{film}$), F_B the buoyancy force ($F_B = gD_0d_1 = gD_{glycerol}d_{film}$), g the gravity constant, D_{film} the density of the film (which will vary according to the amount of disordered nanoscatterers), and $D_{glycerol} = 1258\ kg/m^3$, the density of glycerol.

2. Description of the Monte Carlo approach employed to calculate the optical response of a composite material.

A numerical approach based on the Monte Carlo method that integrates Fresnel coefficients and scattering Mie theory is used to calculate the optical response of inhomogeneous materials. This approach, described in-depth in [35], considers a multilayer system in which each layer is characterized by its thickness (d_m), and the complex refractive index (N_m). Also, layers may contain spherical inclusions of radius (r), volume filling fraction (ff), and complex refractive index (N_{inc}). In this approach, we launch a large number of photons (typically, 10^6 photons for each wavelength λ provides converged results) and track their trajectories from the impingement in the system until the end of their path. When a photon reaches an interface in the multilayer structure, we apply Fresnel theory, and if it reaches an inclusion, we apply Mie's theory taking an absorbing host material [33, 34]. Specifically, the trajectory of a photon is obtained by calculating, consecutively, the distance (l) that the photon travels inside the material before being either scattered by an inclusion or absorbed by any of the comprising materials, and whose expression is the following

$$l = -\ln(p)/\alpha_{ext} \quad (7)$$

being p a random number between 0 and 1, and α_{ext} the extinction coefficient accounting for scattering and absorption of the photon [32-34], i.e,

$$\alpha_{ext} = \alpha_S + \alpha_A \quad (8)$$

Such coefficients are generally defined as:

$$\alpha_S = \rho\sigma_S \quad (13) \text{ and } \alpha_A = \sum_j^M \alpha_{A,j} \quad (9)$$

where ρ is the number density of the scatters, σ_S is the single particle scattering cross-section, and $\alpha_{A,j}$ the absorption coefficient of material j. In the particular case here considered both SiO_2 and PS are absorbers (with $\alpha_A^{PS} = \rho\sigma_A^{PS}$, with σ_A^{PS} the single particle absorption cross-section), while only PS nanospheres scatter light. Therefore:

$$\alpha_S = \rho\sigma_S^{PS} \quad (15) \quad \text{and} \quad \alpha_A = \alpha_{A_2}^{SiO} + \rho\sigma_A^{PS} \quad (10)$$

In particular, the expression of σ_S according to the scattering Mie theory takes the form

$$\sigma_S = \frac{2\pi}{k^2}\sum_{q=1}^{\infty}(2q+1)\left(|a_q|^2 + |b_q|^2\right) \quad (11)$$

where $k = \frac{2\pi}{\lambda \boldsymbol{N}_h}$ is the modulus of the wave vector, and N_h the complex refractive index of the host material. The expansion coefficients a_q and b_q are given by the following expressions:

$$a_q = \frac{m_r \psi_q(m_r x)\psi'_q(x) - \psi_q(x)\psi'_q(m_r x)}{m_r \psi_q(m_r x)\xi'_q(x) - \xi_q(x)\psi'_q(m_r x)} \quad (12) \text{ and } b_q = \frac{\psi_q(m_r x)\psi'_q(x) - m_r \psi_q(x)\psi'_q(m_r x)}{\psi_q(m_r x)\xi'_q(x) - m_r \xi_q(x)\psi'_q(m_r x)} \quad (13)$$

where $\psi_q(\varrho), \psi'_q(\varrho), \xi_q(\varrho)$, and $\xi'_q(\varrho)$ are the Riccati-Bessel functions and their corresponding derivatives respect ϱ. In these expressions, $m_r = N_{inc}/N_h$ stands for the relative complex refractive index of the scattering particle (or inclusion) with respect to the host material, and $x = 2\pi r N_h/\lambda$, is defined as the size parameter, with r the radius of the spherical inclusion.

Lastly, the expression for σ_A is given by

$$\sigma_A = \sigma_{ext} - \sigma_S \quad (14)$$

With

$$\sigma_{ext} = \frac{2\pi}{k^2} \sum_{q=1}^{\infty} (2q+1) Re\{a_q + b_q\} \quad (15)$$

3. Dielectric functions of bulk SiO_2 and PS: tabulated data and fitting with Drude-Lorentz model.

In this work, we apply the Drude-Lorentz model to describe the dielectric function of the materials considered (taking them as if they were homogeneous films). Its expression is given by Eq. (2) in the main manuscript and it assumes that every transition in the material is given by a Lorentz oscillator. With this model, we firstly fit the tabulated data for bulk SiO_2 and PS, which defines the characteristics of the oscillators in the model, and secondly, we use it for determining the dielectric function of the composite thin films, based on the fitted parameters found in the previous step.

Table S1 and S2 gather the fitting parameters for bulk SiO_2 and PS. As an example, Figure S1 shows the fitting curves, in dashed red line, for (a) the imaginary part of the dielectric function (ε''), and (b) the real and imaginary parts of the complex refractive index of bulk SiO_2.

In our approach, the values of ω_j are kept constant for the fitting of the composite thin films made of SiO_2 and PS. In addition, the values of f_j and γ_j are varied in order to minimize the spectral difference among the reflectance, absorptance and transmittance spectra simulated with the Transfer Matrix Method (TMM) using the Drude-Lorentz model for ε_{eff}, and the spectra generated as described in the main manuscript.

SiO$_2$ bulk – fitting parameters

j	ω_j (nm)	f_j(eV)	γ_j(eV)	j	ω_j (nm)	f_j(eV)	γ_j(eV)
1	9	0.006	40.00	*10*	112	0.12	1.00
2	28	0.14	50.00	*11*	118	0.12	0.60
3	45	0.09	15.00	*12*	130	0.01	0.50
4	58	0.09	5.00	*13*	8600	0.04	0.01
5	70	0.11	3.50	*14*	9200	0.14	0.01
6	80	0.11	2.80	*15*	9530	0.12	0.004
7	90	0.11	1.80	*16*	9800	0.15	0.008
8	100	0.11	1.80	*17*	12700	0.10	0.02
9	105	0.12	1.80	*18*	22100	0.85	0.006

Table S1: Fitting parameters of the Drude-Lorentz model (Eq.(2) in the main manuscript) for the dielectric function of bulk SiO$_2$.

PS bulk – fitting parameters

j	ω_j (nm)	f_j(eV)	γ_j(eV)	j	ω_j (nm)	f_j(eV)	γ_j(eV)
1	35	0.0723	40.00	*10*	122	0.07	1.70

2	55	0.1025	11.00	*11*	136	0.07	1.20
3	67	0.12	6.00	*12*	183	0.10	0.90
4	70	0.09	6.00	*13*	190	0.03	0.50
5	79	0.08	2.00	*14*	296.5	0.22	0.49
6	87	0.10	2.00	*15*	213	0.35	0.35
7	96	0.14	2.00	*16*	221	0.015	0.20
8	105	0.12	2.00	*17*	260	0.002	0.50
9	115	0.13	2.00	*18*	3430	0.003	0.01

Table S2: Fitting parameters of the Drude-Lorentz model (Eq.(2) in the main manuscript) for the dielectric function of bulk PS.

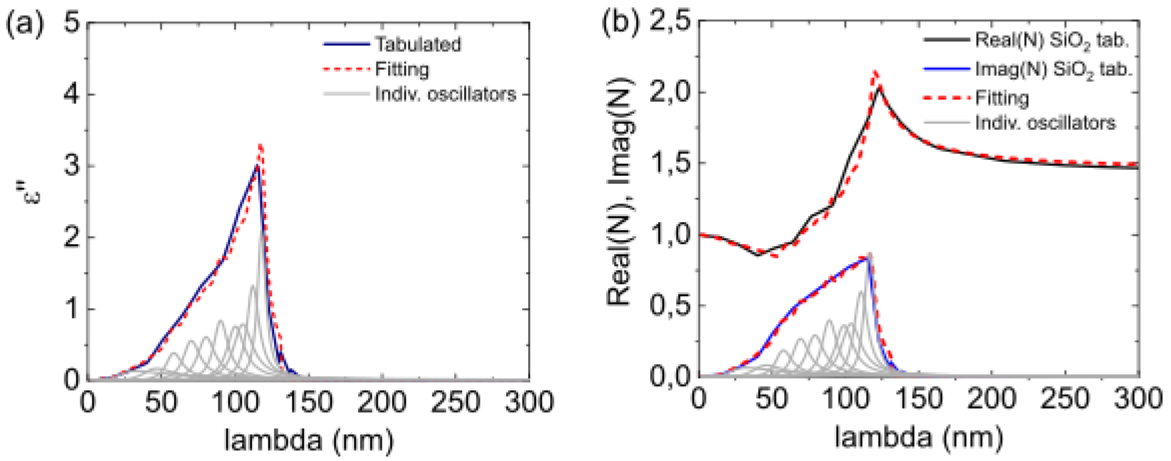

Figure S1: (a) Imaginary part of the dielectric function of bulk SiO_2 as a function of lambda. Solid navy, dashed red, and solid grey lines stand for tabulated, fitted values, and the contribution of each individual oscillator in the model, respectively. (b) Complex refractive index of bulk SiO_2 as a function of lambda. Solid black and blue lines correspond to the real and the imaginary part of the tabulated complex refractive index, while dashed red and grey lines correspond to fitted values and the contribution of each individual oscillator in the model.

4. Maxwell-Garnet effective medium model.

An alternative and most commonly used procedure to describe the optical behavior of a hybrid system is to consider the composite thin film made of a matrix with inclusions of a different material as a homogeneous single layer with an effective dielectric function ($\varepsilon_{eff}(\omega)$). More than ten mixing formulas based on diverse approximations have been proposed in the literature to evaluate $\varepsilon_{eff}(\omega)$ (1), however their accuracy can only be verified by comparison with experiments (2). In particular, for Casimir-Lifshitz calculations, the validation of some of the formulas has been previously analyzed for metallic inclusions in polymeric matrices (3). In this work, we compare our results with those obtained using the widely employed Maxwell-Garnett effective medium model ($\varepsilon_{eff}^{MG}(\omega)$) for spherical inclusions assuming dilute systems (i.e. volume filling fractions (ff) lower than 20%) to discard possible correlation effects. The expression for $\varepsilon_{eff}^{MG}(\omega)$ is the following

$$\varepsilon_{eff}^{MG}(\omega) = \varepsilon_h(\omega)\frac{2(1-ff)\varepsilon_h(\omega)+(1+2ff)\varepsilon_i(\omega)}{(2+ff)\varepsilon_h(\omega)+(1-ff)\varepsilon_i(\omega)} \qquad (S.20)$$

Where $\varepsilon_h(\omega)$ and $\varepsilon_i(\omega)$ are the dielectric functions at frequency ω of the host and the inclusion materials, correspondingly; and ff stands for the volume filling fraction occupied by the inclusion in the host material.

5. Analysis of the optical properties of a 1000 nm thick SiO_2 matrix with ff = 10% of PS particles of diverse size bearing in mind extinction events of photons at λ = 200 nm.

Absorptance (A), percentage of specularly reflected (R_s) and ballistically transmitted (T_b) photons, as well as percentage of diffusively reflected (R_d) and transmitted (T_d) photons at λ = 200 nm impinging on a 1000 nm thick SiO_2 matrix embedding PS particles of diverse size in a 10% volume concentration are shown in Fig. S2 with empty black diamonds, blue stars, and empty black circles. In addition, absorptance of photons occurring after, 1, 2, 3 or 4 particle encounter events is also shown with blue squares, orange circles, yellow upwards triangles, and green downwards triangles, respectively. At λ = 200 nm, bulk SiO_2 does not absorb light, and thus, all absorption in the system must be ascribed to absorption by the PS inclusions. The probability of absorbing a photon in the composite enhances with the number of inclusions (which, for a fixed fill factor, corresponds to smaller particles), as it is observed in the figure. Interestingly, this is reflected in an increment of (R_s + T_b) with the particle size, being the amount of the total diffusely scattered light (R_d + T_d) always $\leq 12\%$, and almost negligible for small particle sizes (r $\leq 40\ nm$).

In addition, results of σ_A and σ_S in Fig. 2 in the main body of the manuscript explain why the probability of absorption occurring after several scattering events increases as the particle size increases, since σ_A and σ_S become of similar intensity. For very small PS inclusions, σ_A >> σ_S. For small particles, the probability of a photon being absorbed in a particle encounter event is

higher than that of being scattered, whereas for larger particles these probabilities tend to be equal, hence the chances of light being absorbed after experiencing diverse scattering events slightly increases. However, in this case, as it was previously mentioned, the number of inclusions in the system is lower, and thus, the probability of a photon to experience an extinction event is reduced, yielding a total absorption lower than that in systems with more particles of smaller size.

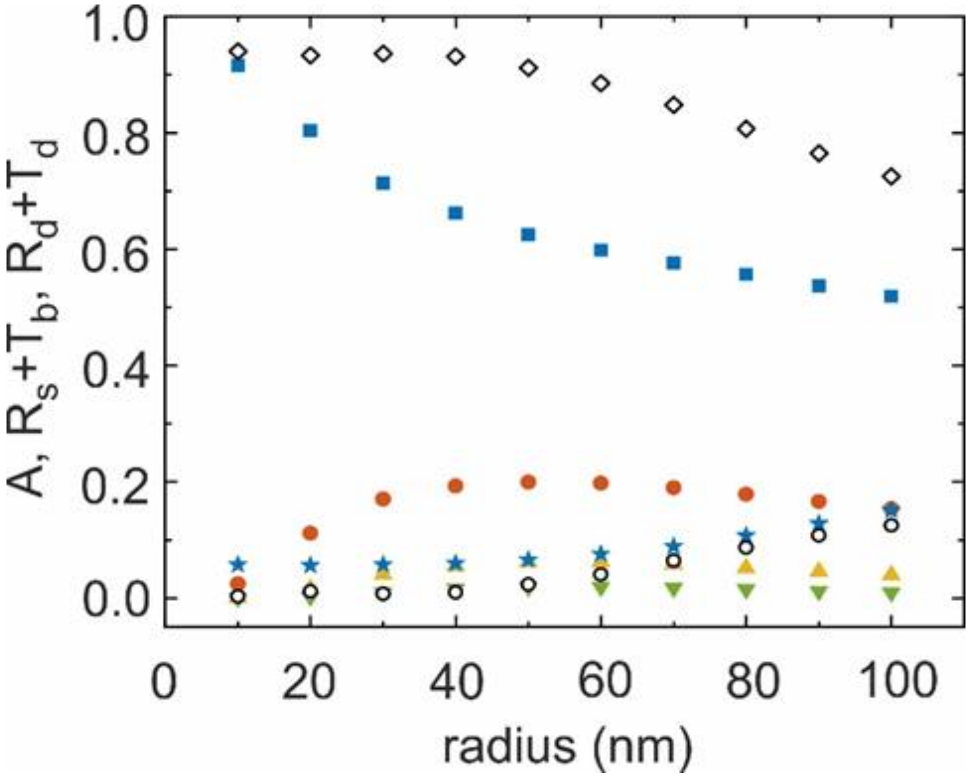

Figure S2. Absorptance (A), percentage of specularly reflected (R_s) and ballistically transmitted (T_b) photons, as well as percentage of diffusively reflected (R_d) and transmitted (T_d) photons at λ = 200 impinging on a 1000 nm thick film made of a SiO_2 matrix with PS spheres (ff = 10 %) as a function of its radius. Empty black diamonds correspond to A, blue stars to (R_s+ T_b), i.e., to the percentage of photons do not suffering any scattering event, and empty black circles to (R_d+ T_d). Blue squares, orange circles, yellow upwards triangles, and green downwards triangles account for absorptance of photons after 1, 2, 3 or 4 particle encounter events, respectively.

References

(1) A. Prasad, K. Prasad, Effective permittivity of random composite media: a comparative study. *Physica B: Condensed Matter*, **2007**, 137, 396

(2) C. G. Granqvist, O. Hunderi, Optical properties of Ag-Si Cermet films: A comparison of effective-medium theories. *Phys. Rev. B*, **1978**, 18, 2897.

(3) R. Esquivel- Sirvent and G. Schatz, Mixing rules and the Casimir force between composite systems, *Phys. Rev. A*, **2011**, 83, 042512
